# Climate Influences Fledgling Sex Ratio and Sex-Specific Dispersal in a Seabird

**DOI:** 10.1371/journal.pone.0071358

**Published:** 2013-08-08

**Authors:** Álvaro Barros, David Álvarez, Alberto Velando

**Affiliations:** Departamento de Ecoloxía e Bioloxía Animal, Universidade de Vigo, Pontevedra, Spain; University of Turku, Finland

## Abstract

Climate influences the dynamics of natural populations by direct effects over habitat quality but also modulating the phenotypic responses of organisms’ life-history traits. These responses may be different in males and females, particularly in dimorphic species, due to sex-specific requirements or constraints. Here, in a coastal seabird, the European shag (*Phalacrocorax aristotelis*), we studied the influence of climate (North Atlantic Oscillation, NAO; Sea Surface Temperature, SST) on two sex-related population parameters: fledgling sex ratio and sex-specific dispersal. We found that fledgling sex ratio was female skewed in NAO-positive years and male skewed in NAO-negative years. Accordingly, females dispersed a longer distance in NAO-positive years when females were overproduced, and on the contrary, males dispersed more in NAO-negative years. Overall, our findings provide rare evidence on vertebrates with genetic sex determination that climate conditions may govern population dynamics by affecting sex-specific density and dispersal.

## Introduction

Climate fluctuations strongly influence the dynamics of natural populations, as revealed by long-term studies [1]–[3]. In many animal species, climate directly affects food and habitat availability, which in turn influence key demographic parameters such as reproductive success [4], [5], survival [6]–[9] and recruitment [10], [11]. Climatic oscillations may also exert complex delayed effects because organisms program phenotypic responses to environmental cues, in many cases, to prepare for the environmental conditions that will encounter during their life [12]. Current evidence suggests that climatic conditions experienced by organisms and cohorts during early development may have carry-over effects that are expressed during adulthood [13], [14] and hence may produce a delayed environment-dependence in population dynamics [15]. For example, in red deer (*Cervus elaphus*) offspring weigth, a key population parameter, is affected by parental weigth at birth, which in turn is governed by climate [16], [17].

Different responses by males and females to climatic oscillations may produce indirect effects on key demographic parameters involved in population dynamics [18]. Thus, for example, changes in temperature may produce bias on primary sex ratios in species with temperature sex determination (e.g. [19], [20]), with important consequences on population dynamics (e.g. [21]). In vertebrates with chromosomal sex determination, the effect of environmental variation on birth sex ratios has also been documented (e.g. [22]–[24]). For instance, in dimorphic species, sex ratio alterations may be produced by sex-specific mortality of the more demanding sex (i.e. the larger sex, which needs more parental supply to be successfully reared) [22] or by maternal production of the more demanding sex when resources are plentiful [25].

Natal dispersal, i.e. movement from birth site to breeding grounds [26], is another key life-history trait with a complex impact on population dynamics [27]. Fluctuations in social and environmental conditions at local natal sites often influence natal dispersal [28]–[30]. Indeed, dispersal may be at least partly the result of adaptive responses to escape negative fitness consequences of conspecific competition and unfavourable environments [26], [31]–[34]. Although the role of climate on natal dispersal has been shown in recent studies (e.g. [35]–[37]), little is known about whether natal dispersal of males and females is similarly affected by climatic oscillations.

In many species, males and females may respond differently to environmental fluctuations because they often differ in their competitive strategies, such as the arrival time to breeding areas [38] and foraging and movement patterns [39]–[42]. Therefore, intraspecific competition is especially intense between same-sex individuals at the local scale [43], [44] and is influenced by variations in the local sex ratio [45], [46]. Thus, intrasexual competition in sex-skewed populations may cause more mortality or emigration in the most abundant sex (e.g. [47]–[49]), although in species in which male harassment is common the opposite trend has been documented [21].

**Figure 1:**
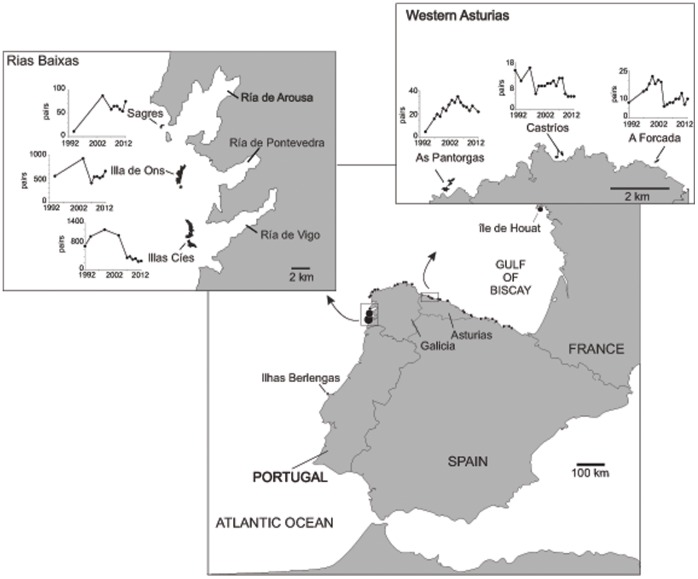
Study area. Distribution of the main colonies (black dots) of the European shag on the Atlantic coast of Spain. The location of the study colonies in two breeding areas are shown in the upper panels. In each colony, the figures on the left hand side show population changes in these colonies between 1992 and 2012; note the differences in scale. The breeding colonies closest to the study area, in Portugal (Ilhas Berlengas) and France (Île de Houat), are shown.

In this study, we evaluated the effect of climate variation on fledgling sex ratio and sex-specific dispersal distance from the natal colony in a long-lived seabird, the European shag (*Phalacrocorax aristotelis*) at the north-west Iberian Peninsula. In particular, we used data from a 21-year-long study carried out in a breeding colony (Illas Cíes) to evaluate the effects of climate variation on fledgling sex ratio. We then used extensive monitoring data from birds marked in six breeding colonies (A Forcada, Castríos, As Pantorgas, Sagres, Illa de Ons and Illas Cíes; Fig. 1) to examine whether climatic variability influences sex-specific dispersal. This dimorphic seabird is an ideal species to study the effects of climate variability on sex-specific life-history traits for several reasons. First, breeding patterns in this coastal seabird are strongly affected by weather and climatic variations [50]–[53]. Second, during the post-fledgling period juveniles compete strongly and males are more aggressive than females [54]. Third, previous evidence suggests that males and females have different competitive strategies [55] and dispersal patterns [56], [57]; indeed, in this species males are constrained by the defence of their breeding territory [58], which may restrict movements during the non-breeding season (e.g. [59]). Fledgling sex ratios are probably affected by food availability [51], and hence by climate oscillation, with possible consequences in sex-specific competence and dispersal patterns.

## Materials and Methods

### Ethics Statement

The work met the Spanish legal requirements about animal welfare and long-term field work was annually supervised and approved by Xunta de Galicia (permit numbers from 1994 to 2012∶3843, 2403, 0103, 1950, 5663, 1301, 1637, 2703, 3004, 12527, 008095, 005453, 2305, 005379, 007434, 1603, 12421, 11380, 002389), Parque Nacional Illas Atlánticas de Galicia (permit number from 2002 to 2012∶3004, 12527, 1902, 1804, 2305, 1604, 1703, 1603, 12421, 1703, 002389) and Principado de Asturias (permit numbers from 1998 to 2012∶000498, 000993, 000775, 001098, 000396, 000688, 000932, 000501, 000779, 001102, 000805, 000698, 000589, 000863, 000665).

### Study Area

This study was carried out in the breeding population of European shag in North-west Iberian Peninsula (Fig. 1). Preliminary analysis of dispersal patterns suggested that this Iberian population is isolated from northern populations [60]. The closest colonies to this population are located 310 km south (Ilhas Berlengas, Portugal) and 500 km north (Île de Houat, Bretagne, France; Fig. 1). Shags generally breed in small colonies (<50 breeding pairs) in the study area, except in the Rías Baixas (Fig. 1) [61], where two main breeding colonies (Illas Cíes and Illa de Ons) accounts for about 70% of the total population in the Atlantic Iberian peninsula [62].

### Fledgling Sex Ratio and Breeding Parameters

The study of fledgling sex ratio was based on data collected between 1992 and 2012 from Illas Cíes, the only breeding colony in the area where such extensive monitoring of breeding parameters was carried out (see [63]). During this period, nest sites were visited three to five times during the breeding season in each year, although in some years (1999, 2010 and 2011) the nesting areas were not monitored. The number of nest monitored varied between 8 (1998) and 93 (1995) due to annual fluctuations in breeding numbers (Fig. 1).

In the monitored areas, chicks were ringed (see below), weighed and measured (bill, head, wing and tarsus lengths), and in some cases a blood sample (about 0.5 ml) was taken from the brachial vein. Chick sex was determined by molecular analysis of blood samples (*n* = 147 chicks) [64], [65] or by means of a discriminant function in chicks older than 25 days (*n* = 193 chicks) [64] when blood samples were not available. In a previous study at Illas Cíes, discriminant function was calculated using 43 chicks sexed molecular analysis; this function correctly sexed over 97% of the chicks [66]. Cohort fledgling sex ratio was calculated as the proportion of males among fledglings (*n* = 9 years). Annual reproductive success (i.e. the number of chicks surviving to full grown, >35 days of age, per nest; [51]) was also monitored in the study areas (*n = *17 years). Laying date of the first egg (*n* = 14 years) was estimated by the earliest egg found by four-day monitoring during the laying period (1994–1996) or by the earliest hatchling in the colony (incubation time 31 days, [66]). In this species, chicks may be accurately aged by the linear relationship between age and wing length during the first weeks of life (wing length = 0.0365*age +1.234; R^2^ = 0.987, [64]). Estimated laying date from earliest hatchling could introduce some inaccuracy if, for example, early pairs have reduced hatching success. Nevertheless, in our population, early pairs have high hatching success (in a three-year study only 6% of early pairs failed to produce a hatchling, [66]), so the error in the estimation of laying date of the first egg was probably minor. Population size was estimated in April-May from direct counts of apparently occupied nests in breeding grounds [62] (see Fig. 1), or it was extrapolated according to available previous and posterior censuses.

### Sex-specific Dispersal

Sex-specific dispersal was analysed by long-term monitoring of marked fledglings in six colonies in two main breeding areas (Fig. 1): Western Asturias (A Forcada, Castríos and As Pantorgas) and the Rías Baixas (Sagres, Illa de Ons and Illas Cíes) in Galicia. A ringing scheme was undertaken in Western Asturias, beginning in 1998 in A Forcada and As Pantorgas and in 1999 in Castríos. In the Rías Baixas, the ringing programme was started in 1992 on Illas Cíes and Illa de Ons, and in 2003 in Sagres. Birds were tagged with a numbered metal ring and a coloured plastic ring with an individual two-digit combination to facilitate identification from distance. In the study areas, nests are clustered in small breeding grounds within the colonies (typically in an area of 100–300 m^2^, thereafter subcolony). Thus, the geographic coordinates of the natal subcolony (i.e. where chicks were ringed) were recorded. Hatching date was estimated from nestling age, calculated by wing length (see above).

During the period 1992–2012, we collected resightings data by intensive field monitoring of marked shags in the studied breeding colonies (all locations were visited three to five times per year). European shags with coloured rings are easily detected during the breeding period and have a high probability of being re-sighted (0.91±0.04 with 95% confidence interval 0.83–0.99, estimated in a previous study from 52 captured-resighted breeding birds [67], [65]). The average age of first reproduction in the study population is 2.53 years [67] and most shags recruit within 3 years [50]. Resightings from throughout the population area, including resightings in the study colonies but also in other minor colonies where birds were not ringed, but which were visited three to seven times along the 10-year study period, and those collected by amateur ornithologists, were used to estimate recruiting distances. The resighting data included 1759 observations of shags ringed as nestlings.

Two-year-old or older ringed birds (i.e. reproductive adults) first observed in breeding colonies were considered recruits (i.e. first time breeders). We sexed 82 of 165 ringed recruits (37 from Western Asturias and 45 from Rías Baixas) by molecular techniques (*n = *23), morphometry (*n = *31) or courtship behaviour (*n = *28). Since European shags move mainly along the coast [58], dispersal distance was estimated as the coastline distance between the natal subcolony and the breeding subcolony in which the bird was first observed using the *measure* tool in Google Earth. When birds recruited in their natal subcolony, dispersal distance was calculated as the half of the coastal distance occupied by the subcolony.

### Annual Climatic Variation

In this study, we used two environmental variables, the North Atlantic Oscillation index (NAO) and the Sea Surface Temperature (SST), as proxies of climatic variation. There is growing evidence that climatic oscillations such as the NAO are regulating forces on marine ecosystems [68]. NAO can be useful to assess the weather severity over a large regional scale [69]–[71], and variation in the NAO index seems to influence population dynamics of seabirds [72]–[74] and marine fish stocks in our study area [75], [76]. NAO data of the average monthly values during the European shag breeding period (February to July) were obtained from the National Oceanographic and Atmospheric Administration of the United States of America (NOAA; ftp://ftp.cpc.ncep.noaa.gov/cwlinks/). On the other hand, SST is also considered as a good proxy for environmental variability at a local scale [53], [79] because it is related to primary marine productivity [77], [78]. SST was estimated for the two breeding areas (Western Asturias and Rías Baixas) from the average monthly values during the breeding period (February to July), obtained from NASA Earth Observations (NEO; http://neo.sci.gsfc.nasa.gov/Search.html). At low latitudes, NAO and the local SST are weakly correlated [80]; indeed, during the studied period (from 1992 to 2012) NAO and local SST did not correlate in both Western Asturias (*r = *0.049, *P* = 0.35; *n* = 21 years) and Rías Baixas (*r* = −0.112, *P* = 0.15; *n* = 21 years), suggesting that these variables reflect different aspects of environmental variation.

### Statistical Analyses

In the Illas Cíes dataset, sex ratio (proportion of male fledglings) was analysed using Generalized Linear Models (GLM) with a binomial error distribution and a logit link function. The dependent variable was the number of fledgling males as numerator and the total number of sexed fledglings as the binomial denominator. In order to avoid over-parametrization, we performed two different bivariate models, one with NAO and the other with SST as an independent variable.

Dispersal distance from six natal colonies in two breeding areas (Western Asturias and Rías Baixas, see Fig. 1) was analysed by using a linear mixed model (LMM), including sex, NAO, SST, breeding area, population size, hatching date, recruiting age and reproductive success as independent variables. Two-ways interactions with sex were also included in the model. Natal colony, nested within breeding area, was included as a random factor. Satterthwaités approximation of degrees of freedom was used [81]. Dispersal distance was log-transformed prior to analysis to achieve error normal distribution. The statistical significance of the random effects was assessed by changes in the likelihood ratio (chi-square log-likelihood) of the model with and without the random effect (Likelihood Ratio Test, LRT). Since the interaction sex*NAO was significant (see results), we also tested whether this effect was similar in both areas by including the triple interaction sex*NAO*breeding area in the final model. All models were simplified by deleting non-significant terms, and the significance level was set at 0.05.

## Results

### Climate Variability and Fledgling Sex Ratio

**Figure 2:**
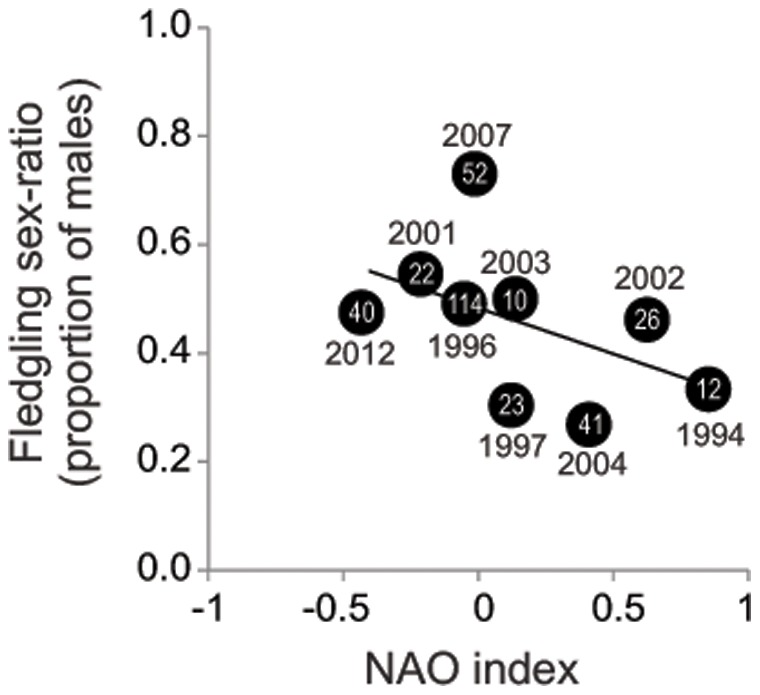
Relationship between sex ratio and NAO index. Proportion of fledging males and NAO index during 1994–2012 on the Illas Cíes. Sample size (number of sexed fledglings) is shown within the circles.

Fledgling sex ratio was negatively related to NAO (estimate = −0.73±0.35; Wald *X* ^2^ = 4.49, *d.f.* = 1, *P = *0.034; *n = *9 years) but not to SST (estimate = 0.36± −0.70; Wald *X* ^2^ = 0.00, d.f. = 1, *P = *0.97; *n = *9 years). In positive NAO years, the sex ratio was female-skewed relative to the ratio in negative NAO years (Fig. 2).

### Climate Variability and Sex-specific Dispersal

**Figure 3:**
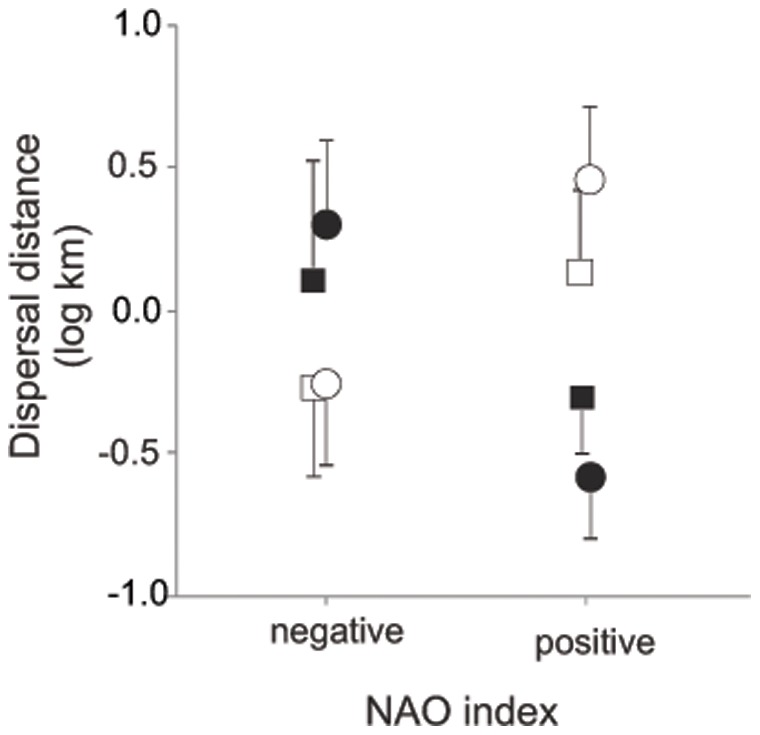
Relationship between natal dispersal distance and NAO index. Distance from natal colony to first breeding colony in the two study areas (see Fig. 1): Western Asturias (squares; *n = *37) and Rías Baixas (circles; *n* = 45). For illustrative purposes, NAO was categorized in positive and negative values. Males are represented with filled symbols and females with open symbols.

Average distance of natal dispersal was 11.53 km (range 0.02 to 318 km) of 82 birds of known sex, which were ringed as chicks (37 males and 45 females). Most of the birds (74.7%) were recruited less than 5 km from their natal site. Overall, the dispersal distances of the sexes were similar (males: 10.59±6.03 km, females: 12.33±7.07 km; *F*_1,79_ = 0.77, *P = *0.38). Climate variability during early development had significant effects on sex-specific patterns of natal dispersal (NAO × sex; Table 1). Thus, females dispersed further in those years with positive values of NAO (slope = 1.28±0.49; *F*_1,43_ = 6.94, *P = *0.012; Fig. 3), but males were recruited from further away when hatched in years with negative values (estimate = −1.34±0.31; *F*_1,36_ = 18.92, *P*<0.0001; Fig. 3).

**Table 1:** Summary of full and minimum adequate general mixed models for dispersal distance in European shags.

Source of variation	Full model				Final model			
	Estimate±SE	*F*	*df*	*P*	Estimate±SE	*F*	*df*	*P*
Intercept	−7.17±7.95				−0.0047±0.14			
Sex (female)	8.85±11.15	0.64	1,70	0.42	−0.15±0.20	0.54	1,78	0.47
Breeding area (Asturias)	0.49±0.46	1.33	1,70	0.25				
NAO	−1.17±0.38	0.10	1,70	0.76	−1.32±0.36	0.01	1,78	0.91
SST	0.48±0.51	0.24	1,70	0.63				
Reproductive success	−0.34±0.30	1.35	1,70	0.25				
Hatching date	0.0021±0.0063	0.11	1,70	0.74				
Recruiting age	0.063±0.054	1.38	1,70	0.24				
Population size	0.00041±0.00037	1.20	1,70	0.28				
NAO*sex	2.52±0.62	16.55	1,70	<0.001	2.57±0.57	20.51	1,78	<0.001
SST*sex	−0.60±0.72	0.68	1,70	0.41				
Breeding area*Sex	−0.077±0.53	0.02	1,70	0.88				
Colony (Breeding area)^a^				0.89				0.89

Data from 82 fledglings.

a Statistical significance of the random factor was analysed by restricted likelihood ratio test.

Breeding area, colony and SST, did not affect dispersal distance (Table 1). The triple interaction sex × NAO × breeding area was not significant when included in the final model (*F*_1,63.1_ = 1.32, *P = *0.25; Fig. 3). The interaction between climate and sex on dispersal distance was also significant when the analysis was restricted to the Illas Cíes (NAO × sex; *F*_1,18_ = 5.26, *P = *0.034).

## Discussion

In this study, we showed that fledgling sex ratio was related to NAO in a breeding colony of European shag. In NAO-positive years, more female fledglings were produced than in NAO-negative years. NAO was also related to natal dispersal, but interestingly this relationship was sex-specific. Females dispersed more widely in NAO-positive years, but on the contrary, male dispersal rate was greatest in NAO-negative years. Overall, these results suggest that climate oscillation influences sex-specific density and dispersal between juveniles.

The results suggest a link between climate oscillation and the variation in fledgling sex ratio in the study area. Females were particularly overproduced in NAO-positive years. There are two alternative, non-exclusive mechanisms, that may explain this result; maternal manipulation of primary sex ratio and sex-specific mortality according to climate conditions. In this dimorphic species, male fledglings are 22% heavier than female fledglings, demanding more parental resources [58] and are probably more sensitive to adverse breeding conditions [64], [82]. Thus, the present results may be explained by climate-dependent food availability (e.g. [83], [84]), if mothers produce more sons when resources are plentiful [25] or by male-specific mortality related to food limitations [85]. In the study area, the abundance of pelagic organisms, such as a fish (*Sardina pilchardus*) and a benthopelagic crab (*Polybius henslowii*), decreased during a NAO-positive phase [75], [76], [86]; future studies should confirm if the abundance of pelagic organisms included in the shag diet are also governed by climatic oscillation. Additionally, NAO-positive values may also be related to nestling mortality [51] due to adverse weather [69]. Finally, variations in NAO may influence the environmental temperature [70], [87], which in turn may influence sex-specific mortality, as shown in other avian species [88]. Independently of the underlying mechanism, the present results suggest the influence of environmental conditions on variations in fledgling sex ratio in a species with chromosomal sex determination.

The present results also indicate that climatic conditions during the nesting stage, as shown by NAO, influenced dispersal distance in the European shag. This result suggests that developmental conditions affect the dispersal from the natal colony to the recruiting place, usually occurring two years later [57]. Thus, in this species early conditions probably influence dispersal decisions of juveniles, with delayed consequences during adulthood, as found in other species [89]–[91]. Indeed, a previous study shown that in the European shag, post-fledgling movements of juveniles are strongly affected by natal environmental [56] and juveniles with greater dispersal distance during their first year of life also breed farther away in their first breeding attempt (our unpublished data). Current evidence suggests that European shags mainly remain during their life-time in the colony where they bred for first time, so the effects of developmental conditions on dispersal maybe long lasting.

Early conditions may modulate dispersal distance from the natal colony via two different mechanisms: by affecting the capacity to disperse or by affecting the motivation to disperse [92] due to the social and non-social (i.e. habitat quality) environment [93]. In the present study, the effect of natal conditions on natal dispersal distance was sex-dependent. This may be explained if, for example, food availability governed by NAO affects the development of flight-capable phenotypes [94]–[97] or if dispersal driven by maternal factors [98], [99] has different effects in males and females. However, we cannot evaluate these possibilities with the available data.

We found that the social environment that juveniles will encounter during the post-fledgling phase was affected by oscillations in climate. Thus, males disperse further in NAO-positive years when more males are produced, and females disperse further in NAO-negative years when more females are produced. Overall, these results suggest that intrasexual competition may at least partly affect dispersal decisions in this species. Young shags may process several sources of information prior to dispersal decisions. During the two months after fledging, juvenile European shags develop social skills and sexual behaviour in crèches around natal colonies [54], [82]. It seems plausible that during this period, individuals will make dispersal decisions depending on the social environment but also in accordance with their competitive abilities [100], [101]. Future studies should evaluate the link between climate oscillation, social environment and behaviour during the post-fledgling period.

In conclusion, we found sex-specific effects of climate variability on two key population parameters in European shags. Thus, NAO during breeding affected fledgling sex ratios and sex-specific natal dispersal distance. These results suggest that climate conditions affect the intrasexual social environment at the natal site, with sex-specific carry-over effects during adulthood, and hence with delayed and prolonged effects on population dynamics. These plastic responses may reveal past adaptations to local environments [14]. Nevertheless, under a scenario of global change, which hypothetically may produce long periods of unidirectional trends in NAO [102], [103], maladaptive long-term effects may occur in this endangered population.
